# Association Between Preoperative Sarcopenia and Postoperative Complications Following Hepatopancreatobiliary Cancer Surgery at a Tertiary Care Center in Eastern India

**DOI:** 10.7759/cureus.101951

**Published:** 2026-01-20

**Authors:** Harshal S Bhoi, Uday Sankar Reddy Kathulapalli, Sumit Mohanty, Satyaprakash Ray Choudhury, Jyotirmay Jena, Swamy H Rajesh, Raghunath Raja M., Dipen Patel, Pratik Bhagat, Adya Panda

**Affiliations:** 1 Department of Surgical Gastroenterology, Institute of Medical Sciences and SUM Hospital, Siksha ‘O’ Anusandhan (Deemed to be University), Bhubaneswar, IND; 2 Department of Radiology, Institute of Medical Sciences and SUM Hospital, Siksha ‘O’ Anusandhan (Deemed to be University), Bhubaneswar, IND

**Keywords:** ct imaging, hepatopancreatobiliary surgery, infection rate, morbidity, postoperative complications, prognostic factors, sarcopenia, skeletal muscle index

## Abstract

Background

Postoperative morbidity continues to be a significant concern in hepatopancreatobiliary (HPB) cancer surgeries despite advances in surgical and perioperative care. Sarcopenia, defined as the loss of skeletal muscle mass and strength, has emerged as an important predictor of poor surgical outcomes. Limited data from India exist regarding its impact on postoperative complications and infections in HPB cancer patients. This study aimed to evaluate the association between preoperative sarcopenia and postoperative morbidity in patients undergoing HPB cancer surgeries.

Methods

A prospective observational study was conducted at the Institute of Medical Sciences and SUM Hospital, Siksha ‘O’ Anusandhan (Deemed to be University), Bhubaneswar, India, between January 2022 and June 2023. Fifty-three patients aged 18-85 years with operable HPB malignancies were included. Sarcopenia was assessed preoperatively using anthropometric measures (mid-arm circumference and handgrip strength) and computed tomography (CT)-derived skeletal muscle index (SMI) at the L3 vertebral level. The cut-off for sarcopenia was defined as <36.5 cm^2^/m^2^ in males and <30.2 cm^2^/m^2^ in females. Postoperative outcomes such as total complications, infective complications, ICU stay, hospital stay, and 30-day mortality were recorded. Statistical analysis was performed using the Mann-Whitney U test, Fisher’s exact test, and univariate logistic regression, with results presented as odds ratios (ORs) and 95% confidence intervals (CIs).

Results

Of the 53 patients, 22 (41.5%) were diagnosed with sarcopenia. No in-hospital mortality or immediate postoperative complications occurred. The total postoperative complication rate was significantly higher in the sarcopenic group (95.45%) compared to the nonsarcopenic group (67.76%; p = 0.014). Infective complications were also more prevalent among sarcopenic patients (45.45%) than nonsarcopenic patients (16.12%; p = 0.019). Univariate analysis identified sarcopenia as a significant predictor of both total postoperative complications (OR = 10.00, p = 0.035) and infective complications (OR = 3.06, p = 0.016).

Discussion

The findings of this study demonstrate that sarcopenia is a key determinant of postoperative morbidity and infection in patients undergoing HPB cancer surgeries. The association between reduced skeletal muscle mass and adverse outcomes may be explained by impaired immune response, reduced metabolic reserve, and delayed tissue recovery in sarcopenic patients. These results are consistent with previous international studies showing higher postoperative complication rates among sarcopenic individuals. Furthermore, the strong correlation between low handgrip strength and infection risk reinforces the importance of assessing both muscle quantity and function in the preoperative setting. The high prevalence of sarcopenia in this study population highlights the need for early recognition, particularly in older adults and those with malignancy-related cachexia.

Conclusion

Sarcopenia is strongly associated with increased postoperative morbidity and infection in HPB cancer surgery. Preoperative identification and targeted prehabilitation strategies focusing on nutrition and muscle strengthening may improve surgical outcomes and enhance recovery in high-risk patients.

## Introduction

Assessment of risk factors prior to any major abdominal surgery is of prime importance to improve outcomes. An important risk factor among them is sarcopenia. Rosenberg introduced the term "sarcopenia," derived from the Greek words “sarx” meaning flesh, and “penia” meaning loss, in the late 1989s [1].

The Asian Working Group for Sarcopenia (AWGS) proposed a definition of sarcopenia as “age-related loss of skeletal muscle mass plus loss of muscle strength and/or reduced physical performance,” and the European Working Group on Sarcopenia in Older People (EWGSOP) defined sarcopenia as the presence of both low muscle mass and low muscle function [2,3]. It can either be primary sarcopenia, i.e., ageing-related or secondary to underlying disease or conditions, such as malnutrition, chronic liver disease, cancer, cardiovascular disease, bone diseases, drugs, particularly long‐term steroid therapy, cytotoxic drugs, etc. [4]. The sarcopenia prevalence in the US and Europe rises from 5% to 13% in patients aged 60-70 years to 11-50% in those more than 80 years. In an Indian study, the prevalence of sarcopenia among individuals were 14.2% in the elderly population above the age of 60 years and higher in females [5]. There is very little awareness about it among health professionals [6].

The postoperative mortality and morbidity after hepatopancreatobiliary (HPB) cancer surgeries remain high, even at high-volume centers. Studies have demonstrated that perioperative complications, infection, mortality, and length of hospital stay after gastrointestinal oncological surgery increase with the presence of sarcopenia. However, few prospective studies have dealt with postoperative outcomes after HPB cancer surgery, focusing on sarcopenia in patients. In this study, we evaluated the association between preoperative sarcopenia and postoperative morbidity and mortality in patients undergoing HPB cancer surgery at a single tertiary care center in Eastern India.

## Materials and methods

This study was approved by the Institutional Ethics Committee, Institute of Medical Sciences and SUM Hospital, Siksha ‘O’ Anusandhan (Deemed to be University), Bhubaneswar (approval no: IEC/IMS.SH/SOA/2022/387; dated 6 July 2022). We performed an observational prospective study of patients who underwent HPB cancer surgeries from January 2022 to June 2023. Inclusion criteria were patients with an age between 18 and 85 years and having operable HPB cancer who gave written informed consent for the study. A patient age less than 18 years and more than 85 years, non-operable HPB cancer, who need neoadjuvant chemotherapy, with connective tissue disorder/severe trauma, and not willing for study were excluded.

Clinical data

After fulfilled the criteria, the enrolled patients were evaluated preoperatively for demographic and clinical factors like age, sex, height, weight, body mass index (BMI), Eastern Cooperative Oncology Group (ECOG) performance status, American Society of Anesthesiologists (ASA) physical status [7], laboratory tests (hemoglobin, total lymphocyte count, serum albumin, tumor markers, etc. were done [8]. For sarcopenia, anthropometric measures like mid-arm circumference (MAC) and the hand grip test for muscle strength were done [9].

Radiological analysis

After the anthropometric and bedside maneuvers, patients who underwent a computed tomography (CT) scan of the abdomen using a 160-slice multidetector CT (MDCT) machine (United Imaging Healthcare Co., Ltd., Shanghai, China) for diagnosis and staging of disease were reevaluated at the level of the third lumbar vertebra (L3) on transverse CT images from each scan, which is universally recognized as a specific method to quantify muscle mass [10]. The CT images were analyzed using manual segmentation with the help of image analysis software Synapse® 3D (FUJIFILM Medical Systems, Tokyo, Japan) [11].

Skeletal muscle area (SMA, cm^2^) was defined as the sum of the paraspinal, psoas, transversus abdominis, internal and external oblique, and rectus abdominis muscles measured at the level of the L3 vertebral endplate. Identification of skeletal muscle and adipose tissue was based on predefined CT attenuation thresholds of -29 to +150 Hounsfield units (HU) for skeletal muscle and -190 to -30 HU for adipose tissue [12]. The total cross-sectional SMA at the L3 level was normalized to height squared (L3 SMA/height^2^) to calculate the L3 skeletal muscle index (SMI, cm^2^/m^2^). Sarcopenia was defined using sex-specific cutoff values of <36.5 cm^2^/m^2^ for males and <30.2 cm^2^/m^2^ for females [13].

All sarcopenic and non-sarcopenic patients underwent the indicated surgical procedures according to their respective malignancies. Postoperatively, patients were assessed for outcomes including length of intensive care unit (ICU) stay and total length of hospital stay. Postoperative complications included pancreatic and biliary leaks, delayed gastric emptying [14], post-pancreatectomy hemorrhage [15], and infections graded according to the Clavien-Dindo classification (grades I-IV) [16]. Postoperative infections, such as specifically wound infection, intra-abdominal abscess, pneumonia, urinary tract infection, and sepsis, were analyzed separately to evaluate the impact of sarcopenia on infectious outcomes. Postoperative mortality was assessed up to 30 days following surgery [17].

Statistical analysis

The collected data were recorded using a structured case record proforma and entered into Microsoft Excel (Microsoft Corp., Redmond, WA, USA), then analyzed using SAS® OnDemand for Academics (SAS Institute Inc., Cary, NC, USA) [18]. Continuous variables were expressed as mean ± standard deviation (SD) or median (interquartile range) as appropriate. Categorical variables were presented as frequencies and percentages. Normality of continuous variables was assessed using the Shapiro-Wilk test [19]. Comparisons between the sarcopenic and non-sarcopenic groups were performed using the Mann-Whitney U test for non-normally distributed continuous variables and the independent sample t-test for normally distributed data [20]. The chi-square test or Fisher’s exact test was used to compare categorical variables [21]. Univariate logistic regression analysis was performed to identify potential predictors of total postoperative complications and postoperative infective complications. Odds ratios (ORs) with 95% confidence intervals (CIs) were calculated to determine the strength of association between each variable and postoperative outcomes [22]. Variables with p < 0.10 in univariate analysis were included in multivariate logistic regression models to identify independent predictors. A p-value < 0.05 was considered statistically significant for all analyses.

## Results

A total of 53 patients who underwent HPB surgery for malignancy were included in this study. Sarcopenia was determined based on CT-derived SMI values. No in-hospital mortality or immediate postoperative complications (within 24 hours) were recorded. The demographic and clinicopathological characteristics of the study population are shown in Table 1. Among the study population, 54.7% were males and 45.3% were females. Sarcopenia was present in 22 patients (41.5%), while 31 patients (58.5%) were classified as non-sarcopenic. The mean age of the sarcopenic group was 58.59 years, which was slightly higher than that of the non-sarcopenic group (54.16 years). The mean SMI values for non-sarcopenic patients were 43.2 ± 1.49 cm^2^/m^2^ in males and 36.92 ± 0.96 cm^2^/m^2^ in females, whereas sarcopenic patients showed lower values of 34.09 ± 1.01 cm^2^/m^2^ and 27.62 ± 0.86 cm^2^/m^2^, respectively. The overall mean SMI of the non-sarcopenic group (40.32 ± 3.38 cm^2^/m^2^) was significantly higher than that of the sarcopenic group (31.15 ± 3.42 cm^2^/m^2^; p < 0.001). In addition, the MAC and handgrip strength were significantly reduced among sarcopenic patients (p < 0.001 and p = 0.011, respectively), indicating a clear difference in muscle mass and strength between the groups.

**Table 1:** Demographic and Clinicopathological Characteristics of the Study Population An independent t-test was used to test the statistical significance. P-values <0.05 were considered significant. BMI: body mass index; MAC: mid-arm circumference; SMI: skeletal muscle index

Variables	Nonsarcopenic (n = 31)	Sarcopenic (n = 22)	t-value	p-value
Male/Female	17/14	12/10	-	-
Mean Age (years)	54.16 ± 12.6	58.59 ± 11.2	-1.347	0.1844
Height (cm)	160.48 ± 9.22	160.31 ± 9.33	0.066	0.9479
Weight (kg)	59.00 ± 8.76	59.23 ± 8.85	-0.094	0.9258
BMI (kg/m^2^)	22.79 ± 1.40	22.95 ± 1.54	-0.387	0.7008
MAC (cm)	29.69 ± 4.70	24.86 ± 4.90	3.596	0.0008
Handgrip Strength (kg)	39.21 ± 9.50	32.21 ± 9.30	2.676	0.0103
SMI in Males [23]	43.12 ± 1.49	34.09 ± 1.01	26.289	0.0054
SMI in Females [23]	36.92 ± 0.96	27.62 ± 0.86	36.95	0.0048
Mean Blood Loss (mL)	530 ± 110	582 ± 160	-1.319	0.1958
Mean Operative Time (min)	310 ± 56	322 ± 80	-0.606	0.5484

The comparison of biochemical parameters between the two groups revealed no statistically significant differences. Mean hemoglobin levels were slightly higher in sarcopenic patients (11.17 ± 1.31 g/dL) compared to nonsarcopenic patients (10.71 ± 1.90 g/dL; p = 0.341). Similarly, serum albumin levels showed a nonsignificant trend toward higher values in the sarcopenic group (3.97 ± 0.77 g/dL vs. 3.74 ± 0.81 g/dL; p = 0.302). Total and direct bilirubin levels were also higher among sarcopenic individuals, though without statistical significance (p = 0.236 and p = 0.186, respectively). No in-hospital mortality or immediate postoperative complications (within 24 hours) were observed, indicating comparable short-term postoperative outcomes between sarcopenic and nonsarcopenic patients. The demographic variables, such as age, sex, height, weight, and BMI, and laboratory parameters, such as hemoglobin, albumin, and bilirubin, were comparable between both groups. The laboratory findings are summarized in Table 2.

**Table 2:** Laboratory Values of Nonsarcopenic and Sarcopenic Patients An independent t-test was used to test the statistical significance. P-values <0.05 were considered significant.

Variables	Nonsarcopenic (n = 31)	Sarcopenic (n = 22)	t test	p-value
Hemoglobin (g/dL)	10.71 ± 1.90	11.17 ± 1.31	-1.043	0.341
Serum Albumin (g/dL)	3.74 ± 0.81	3.97 ± 0.77	-1.049	0.302
Serum Total Bilirubin (mg/dL)	1.48 ± 1.90	2.90 ± 6.10	-1.056	0.236
Serum Direct Bilirubin (mg/dL)	0.98 ± 1.55	2.21 ± 4.70	-1.183	0.186

Comorbidities such as hypertension, diabetes, chronic kidney disease, pacemaker use, and hypothyroidism are presented in Table 3, with no significant differences observed between the groups. ASA physical status, ECOG score, and disease etiology were also similar. The surgical procedures performed included pancreatoduodenectomy (n = 33), radical cholecystectomy (n = 16), hepatectomy (n = 2), and distal pancreaticosplenectomy (n = 2). The mean blood loss and operative time were comparable between groups, with 582 ± 160 mL and 322 ± 80 minutes in sarcopenic patients, and 530 ± 110 mL and 310 ± 56 minutes in non-sarcopenic patients, respectively.

**Table 3:** Frequencies of Comorbidities in the Study Population

Comorbidities	Nonsarcopenic (n = 31)	Sarcopenic (n = 22)
Hypertension	0	2 (9.09%)
Any Pacemaker Usage	1 (3.22%)	0
Diabetes Mellitus	5 (16.12%)	1 (4.54%)
Chronic Kidney Disease (CKD)	1 (3.22%)	0
Hypothyroidism	2 (6.45%)	0

Postoperative outcomes were compared between nonsarcopenic (n = 31) and sarcopenic (n = 22) patients who underwent HPB surgery for malignancy, as shown in Table 4. There was no postoperative mortality in either group. However, total postoperative complications were significantly higher among sarcopenic patients (95.45%) compared to nonsarcopenic patients (67.76%; p = 0.014). Similarly, postoperative infective complications were more frequent in the sarcopenic group (45.45%) than in the nonsarcopenic group (16.12%; p = 0.019). The mean ICU stay was slightly longer in sarcopenic patients (2.27 ± 1.38 days) than in nonsarcopenic patients (1.87 ± 1.14 days), though the difference was not statistically significant (p = 0.255). Likewise, the average duration of hospital stay tended to be longer in the sarcopenic group (11.73 ± 7.20 days) compared to the nonsarcopenic group (9.19 ± 3.09 days), but this did not reach statistical significance (p = 0.087).

**Table 4:** Postoperative Short-Term Outcomes The χ^2^ test was used for total postoperative complications and postoperative infective complications; the Mann-Whitney U test was used to test the statistical significance for ICU stay and hospital stay. P-values <0.05 were considered significant. * indicates p-value < 0.05 showing statistical significance.

Variable	Nonsarcopenic (n = 31)	Sarcopenic (n = 22)	Test Statistic	p-value
Mortality	0	0	-	-
Total Postoperative Complications	21 (67.76%)	21 (95.45%)	χ^2^ = 4.44	0.035*
Postoperative Infective Complications	5 (16.12%)	10 (45.45%)	χ^2^ = 4.10	0.043*
ICU Stay (days)	1.87 ± 1.14	2.27 ± 1.38	U = 291.0	0.372
Hospital Stay (days)	9.19 ± 3.09	11.73 ± 7.20	U = 301.0	0.476

Univariate analysis was performed to identify preoperative factors associated with total postoperative complications in patients undergoing cancer surgery, as shown in Table 5. Among the assessed variables, sarcopenia emerged as the only significant predictor of postoperative complications (p = 0.035), with an OR of 10.00 (95% CI: 1.174-85.21), indicating that sarcopenic patients were 10 times more likely to experience complications compared to nonsarcopenic individuals. Other factors such as age, sex, hemoglobin, serum albumin, BMI, MAC, handgrip strength, ASA grade, ICU stay, and hospital stay did not show statistically significant associations with postoperative complications (p > 0.05). Although trends were observed with lower MAC and higher BMI potentially increasing complication risk (OR = 2.84 and 4.10, respectively), these did not reach significance. Overall, the findings highlight sarcopenia as a key preoperative risk factor influencing postoperative morbidity in patients undergoing HPB malignancy surgery.

**Table 5:** Univariate Regression Analysis of Preoperative Factors Associated With Total Postoperative Complications in Patients Undergoing HPB Cancer Surgery HPB: hepatopancreatobiliary; BMI: body mass index; MAC: mid-arm circumference; ASA: American Society of Anesthesiologists (Physical Status Classification); ICU: intensive care unit

Factors	Odds Ratio (OR)	95% Confidence Interval (CI)
Age (≥50 vs. <50 years)	0.40	0.076-2.10
Sex (Male vs. Female)	1.01	0.266-3.83
Hemoglobin (>10 vs. <10 g/dL)	0.68	0.155-2.93
Serum Albumin (>3.5 vs. <3.5 g/dL)	1.27	0.317-5.13
BMI (<25 vs. ≥25 kg/m^2^)	4.10	0.236-71.4
MAC (<24.86 vs. ≥24.86 cm)	2.84	0.717-11.27
Handgrip Strength (<32.21 vs. ≥32.21 kg)	1.01	0.266-3.83
ASA Grade (I vs. II) [7]	1.26	-
Sarcopenia (Yes vs. No)	10.00	1.174-85.21
ICU Stay (>3 vs. <3 days)	1.30	-
Hospital Stay (>8 vs. <8 days)	1.95	0.510-7.45

Univariate analysis of preoperative factors associated with postoperative infective complications in patients undergoing HPB surgery revealed that sex, handgrip strength, sarcopenia, and hospital stay were significantly associated with infection risk, as depicted in Table 6. Male patients demonstrated a higher likelihood of developing infective complications compared to females (p = 0.032; OR = 5.37; 95% CI: 1.15-25.03). Reduced handgrip strength was found to be a protective factor, with patients having higher strength showing significantly lower risk of infection (p = 0.020; OR = 0.21; 95% CI: 0.06-0.78). Sarcopenia was also significantly associated with infective complications (p = 0.016; OR = 3.07; 95% CI: 0.64-14.74), suggesting increased susceptibility among sarcopenic individuals. Moreover, longer hospital stays (>8 days) were linked to reduced odds of infection (p = 0.035; OR = 0.20; 95% CI: 0.04-0.90), possibly reflecting prolonged care and antibiotic coverage. Other variables, including age, hemoglobin, albumin, BMI, MAC, ICU stay, and ASA grade, were not significantly associated with postoperative infections.

**Table 6:** Univariate Regression Analysis of Preoperative Factors Associated With Postoperative Infective Complications in Patients Undergoing HPB Surgery HPB: hepatopancreatobiliary; BMI: body mass index; ASA: American Society of Anesthesiologists (Physical Status Classification); ICU: intensive care unit

Factors	Odds Ratio (OR)	95% Confidence Interval (CI)
Sex (Male vs. Female)	5.37	1.15-25.03
Age (≥50 vs. <50 years)	0.88	0.17-4.53
Hemoglobin (>10 vs. <10 g/dL)	1.53	0.31-7.47
Serum Albumin (>3.5 vs. <3.5 g/dL)	0.60	0.11-3.18
BMI (≥25 vs. <25 kg/m^2^)	7.10	-
Mid-Arm Circumference (MAC) (<24.82 vs. ≥24.82 cm)	0.76	0.24-2.41
Handgrip Strength (<32.21 vs. ≥32.21 kg)	0.21	0.06-0.78
Sarcopenia (Yes vs. No)	3.07	0.64-14.74
ICU Stay (>3 vs. <3 days)	4.83	-
Hospital Stay (>8 vs. <8 days)	0.20	0.04-0.90
ASA Grade (II vs. I) [7]	0.74	0.06-9.67

A comparison of postoperative complication rates between sarcopenic and nonsarcopenic patients across different studies is summarized in Table 7. In the present study, postoperative complications were significantly higher among sarcopenic patients (95.45%) compared to nonsarcopenic patients (67.76%; p = 0.014), indicating a strong association between sarcopenia and adverse surgical outcomes following HPB surgery. Similar findings were reported across multiple studies evaluating the impact of sarcopenia on postoperative outcomes. Van Rijssen et al. [24] observed postoperative complication rates of 66.9% in sarcopenic patients and 63.9% in nonsarcopenic patients following pancreaticoduodenectomy, with no statistically significant difference (p = 0.73). In contrast, Kitano et al. [25] reported a significantly higher incidence of postoperative complications among sarcopenic individuals (71%) compared to nonsarcopenic counterparts (50.6%; p = 0.04) in patients undergoing surgery for extrahepatic cholangiocarcinoma. Similarly, Choi et al. [26] found higher complication rates in sarcopenic patients (60%) versus nonsarcopenic patients (50%) following pancreatic cancer surgery, though the difference did not reach statistical significance (p = 0.438). These inter-study variations may stem from differences in patient selection, underlying malignancy types, surgical complexity, and heterogeneity in sarcopenia definitions or measurement techniques. Nonetheless, the overall trend consistently demonstrates that sarcopenic patients experience poorer postoperative outcomes. Collectively, the evidence reinforces sarcopenia as an independent and clinically meaningful predictor of postoperative morbidity in major abdominal and HPB surgeries. Integrating sarcopenia screening into preoperative assessments could therefore help identify high-risk individuals and guide tailored interventions to improve surgical safety and recovery.

**Table 7:** Comparison of Postoperative Complications Between Sarcopenic and Nonsarcopenic Patients in Different Studies

Study	Postoperative Complications (Sarcopenia)	Postoperative Complications (Nonsarcopenia)	χ^2^ Value	p-value
Present Study	95.45%	67.76%	22.42	0.014
Van Rijssen et al. [24]	66.9%	63.9%	0.09	0.73
Kitano et al. [25]	71%	50.6%	7.59	0.04
Choi et al. [26]	60%	50%	1.64	0.438

## Discussion

The present study investigated the impact of sarcopenia on postoperative morbidity and infection in patients undergoing HPB surgery for malignancy. Among 53 eligible patients, no in-hospital mortality or immediate complications were reported. In postoperative outcomes, ICU stay was similar between groups; however, the sarcopenia group had a longer mean hospital stay (p = 0.087). While no immediate complications were seen, total late postoperative complications were significantly higher in sarcopenic patients (95.45%) than in non-sarcopenic ones (67.76%; p = 0.0142). Comparable findings were observed in other studies: Van Rijssen et al. reported 66.9% vs. 63.9% morbidity post-pancreaticoduodenectomy (p = 0.73) [24]; Kitano et al. [25] found 71% vs. 50.6% complications in extrahepatic cholangiocarcinoma (p = 0.04) [25]; and Choi et al. reported 60% vs. 50% for pancreatic cancer (p = 0.438) [26]. Overall, these results underscore a consistent trend toward higher postoperative morbidity among sarcopenic patients. In univariate analysis, sarcopenia was associated with a 10-fold higher risk of postoperative complications (p = 0.035), highlighting its clinical relevance as a preoperative prognostic factor. Males comprised 54.7% of the study population, showing a slight predominance over females (45.3%), consistent with earlier reports highlighting a higher incidence of HPB malignancies in men (Li et al., 2019 [27]; Miller, 2021 [28]). The mean age was 58.59 years in the sarcopenia group and 54.16 years in the non-sarcopenia group (p = 0.196), aligning with studies linking sarcopenia and advancing age (Fuggle et al., 2017 [29]; Cruz-Jentoft and Sayer, 2019 [30]). Sarcopenia prevalence was 41.5%, higher than general Indian estimates (14.2-17.5%) (Tyrovolas et al., [31]), but similar to rates in HPB malignancies such as pancreatic (30-65%) and hepatic cancers (40-55%) (Chan et al., [32]; Peng et al. and Peng et al. [33,34]; Voron et al., [35]). Significant differences were observed in MAC (p < 0.001) and hand grip strength (p = 0.011), while other baseline factors (sex, BMI, comorbidities, laboratory variables) showed no significance. The mean SMI was markedly lower in sarcopenic patients (31.15 vs. 40.32; p < 0.001), confirming SMI’s diagnostic validity (Martin et al., 2013 [36]; Cruz-Jentoft and Sayer, 2019 [30]).

Regarding infectious complications, the sarcopenia group exhibited a significantly higher infection rate (45.45%) compared to the non-sarcopenia group (16.12%; p < 0.019). This aligns with Takagi et al., who observed increased infective complications in sarcopenic patients undergoing HPB surgeries (36.8% vs. 17.2%; p = 0.015) [17]. Males were also at greater risk, with 5.37 times higher odds of infection than females (p = 0.032), while prolonged hospital stay (>8 days) correlated with lower infection odds (p = 0.035). Additionally, hand grip strength showed a significant inverse association with infection risk (p = 0.020; OR = 0.214). The elevated susceptibility to infections in sarcopenic patients may stem from impaired immune function and delayed wound healing, emphasizing the need for preoperative nutritional and physical optimization in HPB cancer surgery candidates.

Limitations

This study has several limitations that warrant consideration. First, the relatively small sample size (n = 53) may limit the statistical power and generalizability of the findings. Second, being a single-center study, institutional practices, surgical techniques, and perioperative care protocols may have influenced outcomes, potentially introducing selection bias. Third, sarcopenia was assessed only through CT-derived SMI, without evaluating muscle quality or fat infiltration, which are known to affect functional outcomes. Additionally, the study did not account for confounding variables such as nutritional status, inflammatory markers, or tumor stage, which may independently impact postoperative morbidity. Lastly, follow-up was limited to the in-hospital period, restricting insights into long-term outcomes such as survival or recurrence.

Future directions

Future research should focus on multicenter prospective studies with larger cohorts to validate these findings across diverse populations and surgical subtypes of HPB malignancies. Incorporating comprehensive assessments, such as muscle attenuation, bioelectrical impedance, or dual-energy X-ray absorptiometry (DEXA), could enhance diagnostic accuracy for sarcopenia. Future studies should also evaluate the role of prehabilitation strategies, including nutritional supplementation, resistance training, and physiotherapy, in mitigating postoperative complications. Moreover, integrating inflammatory and metabolic biomarkers may help elucidate the mechanistic links between sarcopenia and postoperative morbidity. Long-term follow-up evaluating survival, quality of life, and functional recovery will provide deeper insight into the prognostic value of sarcopenia and guide tailored perioperative interventions in HPB cancer patients.

## Conclusions

In conclusion, this study highlights the critical role of preoperative sarcopenia assessment in patients undergoing HPB cancer surgery. Sarcopenia was significantly associated with higher rates of total and infective postoperative complications, emphasizing its impact on surgical recovery and patient outcomes. Identifying sarcopenic patients before surgery provides an opportunity for clinicians to implement targeted prehabilitation strategies focusing on nutritional optimization, resistance training, and metabolic support to enhance muscle mass and functional reserve. Such proactive interventions could improve postoperative resilience, reduce complication rates, and ultimately enhance the overall well-being and quality of life of patients undergoing major HPB procedures. These findings underscore the need to integrate sarcopenia screening as a standard component of preoperative evaluation, transforming surgical planning from a reactive to a preventive approach. By addressing sarcopenia early, healthcare teams can move toward personalized, outcome-oriented perioperative care in HPB oncology.
